# Evaluating the Additional Value of Endoscopic Ultrasonography for Depth Assessment of Esophagogastric Junction Adenocarcinoma

**DOI:** 10.1002/deo2.70215

**Published:** 2025-09-30

**Authors:** Keita Suzuki, Yohei Ikenoyama, Ken Namikawa, Yoshitaka Tokai, Shoichi Yoshimizu, Yusuke Horiuchi, Akiyoshi Ishiyama, Toshiyuki Yoshio, Toshiaki Hirasawa, Manabu Takamatsu, Takahisa Matsuda, Junko Fujisaki

**Affiliations:** ^1^ Department of Gastroenterology Cancer Institute Hospital, Japanese Foundation for Cancer Research Tokyo Japan; ^2^ Division of Gastroenterology and Hepatology Department of Internal Medicine Toho University, Omori Medical Center Tokyo Japan; ^3^ Department of Gastroenterology and Hepatology Mie University Hospital Mie Japan; ^4^ Division of Pathology Cancer Institute Hospital, Japanese Foundation for Cancer Research Tokyo Japan

**Keywords:** adenocarcinoma, cancer, endoscopy, esophagogastric junction, ultrasonography

## Abstract

**Objectives:**

Endoscopic ultrasonography (EUS) is sometimes used to assess the depth of invasion in esophagogastric junction adenocarcinoma (EGJA); however, its diagnostic performance in EGJA remains unclear. This study aimed to evaluate the additional value of EUS to conventional endoscopy (CE) in assessing invasion depth.

**Methods:**

In this single‐institution retrospective study, we compared the diagnostic performance of CE alone with that of CE + EUS for preoperative depth assessment of early‐stage EGJA. In addition, we examined the clinicopathologic features associated with incorrect depth assessment.

**Results:**

The study included 93 cases of early‐stage EGJA. Comparing the diagnostic performance for diagnosing submucosal cancer (CE vs. CE + EUS), CE + EUS had a significantly lower specificity than CE alone (78.4% vs. 62.2%). However, no significant differences were observed in sensitivity (73.2% vs. 71.4%) and accuracy (75.3% vs. 67.7%) between the two modalities. The addition of EUS was associated with significantly higher misdiagnosis rates in the following types of lesions: lesions located on the esophageal side (42.6% vs. 25.5%), elevated lesions (29.2% vs. 15.4%), complex lesions (32.7% vs. 16.3%), and lesions with hiatal hernia (31.1% vs. 19.7%). No clinicopathological factors were significantly associated with overdiagnosis or underdiagnosis.

**Conclusions:**

The addition of EUS to CE reduced the ability to accurately identify mucosal cancers in early‐stage EGJA, suggesting a risk of overdiagnosis and unnecessary therapeutic escalation.

## 1 Introduction

Esophagogastric junction adenocarcinoma (EGJA) is a relatively rare disease; however, its incidence has been on the rise in recent years, providing increased opportunities for treatment [1, 2, 3]. The frequency of lymph node metastasis in gastrointestinal cancers is strongly correlated with the depth of invasion [4, 5], with significant differences between endoscopic treatment and surgical resection in terms of degree of invasiveness for patients [6, 7]. Therefore, preoperative assessment of the depth of cancer invasion plays a pivotal role in determining the appropriate treatment strategy. Typically, gastrointestinal cancers require a comprehensive depth assessment that combines multiple modalities.

Endoscopic ultrasonography (EUS) has long been considered effective for depth assessment in the upper gastrointestinal tract [8, 9, 10, 11]. However, the assessment of the depth of invasion in esophagogastric junction cancers tends to be more difficult than that in esophageal and gastric cancers outside of the junctional region, and the utility of EUS is controversial [12].

At our institution, we have adopted a practice of supplementing conventional endoscopy (CE) with EUS, primarily in cases where depth assessment is difficult with the use of CE alone. In the present study, we aimed to explore the feasibility of combining EUS with CE to assess invasion depth in cases of early EGJA with mucosal (M) to submucosal (SM) invasion.

## 2 Methods

### 2.1 Patients and Study Design

In this single‐institution retrospective study, a total of 93 patients with early‐stage EGJA who underwent both CE and EUS between January 2012 and December 2022 were included. The diagnostic performances of CE and CE + EUS for invasion depth assessment in early‐stage EGJA were evaluated by comparison with the final pathological findings.

As diagnostic specificity was considered the primary outcome due to its clinical relevance in avoiding overtreatment, our analysis primarily focused on specificity. In addition, we examined the clinicopathological features associated with misdiagnosis, including overdiagnosis and underdiagnosis.

### 2.2 Definitions of Esophagogastric Junction and Adenocarcinoma of the Esophagogastric Junction

The esophagogastric junction (EGJ) is endoscopically defined as “the distal ends of esophageal longitudinal palisade vessel.” In cases where identifying the palisade vessel proves challenging, it is alternatively defined as “the proximal ends of gastric longitudinal mucosal folds,” which is the common definition in Europe and the United States [13]. EGJA was defined as a type II junctional adenocarcinoma based on the Siewert classification and characterized by the tumor center (the deepest part) being located within 1 cm above and 2 cm below the EGJ [14, 15].

### 2.3 Clinical and Endoscopic Evaluation

The clinical characteristics of patients, such as age, sex, body mass index (BMI), and the Brinkman index, were extracted from medical records. All endoscopic procedures were performed by endoscopists affiliated with the Japan Gastroenterological Endoscopy Society.

For CE, the EVIS LUCERA SPECTRUM, EVIS LUCERA ELITE, EVIS X1 (Olympus, Tokyo, Japan), and the GIF‐H260Z, GIF‐H290Z, and GIF‐XZ1200 (Olympus, Tokyo, Japan) endoscopes were used. Depth of invasion diagnoses using CE were primarily based on white light imaging observations, estimated by evaluating the lesion's size, thickness, morphological findings, and shape alterations resulting from the volume of insufflated air. As findings indicative of SM invasion included type 0‐I lesions ≥20 mm in size, macroscopic complex types, and lack of shape alteration of the lesion upon deflation [16, 17]. Although magnifying image‐enhanced endoscopy (IEE) was used in some cases, specific findings could not be consistently extracted from the retrospective reports; therefore, they were excluded from the analysis.

EUS was performed using the UM‐3R‐20‐MHz ultrasound endoscope (Olympus, Tokyo, Japan) with the EU‐M2000 endoscopic ultrasound system (Olympus, Tokyo, Japan) and the de‐aerated water immersion method for imaging. In some cases involving thick tumors, the GF‐UM20‐7.5/12‐MHz (Olympus, Tokyo, Japan) was used. EUS reveals that the normal EGJ is composed of five distinct layers [12]_._ The following criteria were used for cancer depth assessment: 1) tumor is clinically confined within the epithelial/intrinsic layer, indicating that the third layer is uninterrupted (M) and 2) tumor clinically invades the submucosal layer, indicating that the third layer is irregular and interrupted (SM invasion). Information regarding invasion depth assessed using both CE and EUS was retrospectively extracted from the endoscopic reports recorded at the time of examination. In cases where CE and EUS findings were discordant, the final depth diagnosis and treatment decision were made based on a comprehensive clinical evaluation, considering all available information.

The localization of each tumor was classified as follows: (i) the central part of the tumor was primarily located on the esophageal side ≥ gastric side or gastric side > esophageal side and (ii) the tumor's lateral position was identified as either the right or left side, based on the forward EGJ view. Lesion circumference was classified as either <1/2 or ≥1/2. The main macroscopic types were classified according to the Paris endoscopic classification [18] and divided according to the following two criteria: (i) into two groups according to main macroscopic type: elevated (0‐I, 0‐IIa) and flat‐depressed (0‐IIb, 0‐IIc) and (ii) into simple (elevated, flat‐depressed) and complex types (mixed elevated and flat‐depressed). In addition, the presence or absence of gastroesophageal reflux disease (GERD) and hiatal hernias of the esophagus were examined.

### 2.4 Histopathological Evaluation

Histopathological examination of specimens resected via endoscopic treatment and surgical procedures was conducted using hematoxylin and eosin staining. Sections were prepared with thicknesses of 2 and 5 mm for endoscopically and surgically resected specimens, respectively. Tumor differentiation and invasion depth were evaluated according to the Japanese classification [13, 15]. The degree of differentiation was classified based on the presence or absence of undifferentiated components. In this study, “undifferentiated” was defined as poorly differentiated adenocarcinoma or signet‐ring cell carcinoma. The depth was classified into two groups: M and SM. The final histopathological evaluations were compiled from the pathologist's original reports.

### 2.5 Outcome Measures

To reduce risks of overtreatment owing to depth misdiagnosis in EGJA, our study focused on diagnostic specificity as the primary outcome measure. This approach aimed to decrease unnecessary invasive treatments for cases inaccurately diagnosed with SM invasion. In addition, maintaining high sensitivity is crucial for the accurate detection of true SM invasions, thus facilitating appropriate patient management.

Consequently, the study's outcome measures were defined as follows, aiming to evaluate the advantages of combining EUS with CE for invasion depth assessment:

- Primary outcome:
- Specificity, defined as the proportion of cases preoperatively assessed as M that were confirmed via pathological assessment to be M.
- Secondary outcomes:
- Sensitivity, defined as the proportion of cases preoperatively assessed as SM that were confirmed via pathological assessment to be SM.

Accuracy, defined as the number of all cases (M and SM) correctly diagnosed preoperatively and confirmed via pathological assessment, divided by the total number of cases.

In addition, as a supplementary indicator of diagnostic stability for depth assessment, we analyzed the clinicopathological factors associated with overall misdiagnosis. Furthermore, as exploratory analyses, we examined the clinicopathological factors related to overdiagnosis and underdiagnosis. The overdiagnosis rate was defined as the proportion of cases clinically diagnosed as SM invasion but pathologically confirmed as M, whereas the underdiagnosis rate was defined as the proportion of cases clinically diagnosed as M but pathologically confirmed as SM.

### 2.6 Statistical Analysis

Statistical analyses were performed using EZR, version 1.61 (Saitama Medical Center, Jichi Medical University, Saitama, Japan). Quantitative data were presented as medians and interquartile ranges, as appropriate. Fisher's exact test was used for comparisons between independent groups, and McNemar's test was applied for paired comparisons. A two‐tailed *p*‐value <0.05 indicated statistical significance.

## 3 Results

### 3.1 Patient and Lesion Characteristics

The clinicopathological characteristics of the included patients and lesions are shown in Table 1. The median age of the patients was 67 [interquartile range (IQR), 58–74] years; the median BMI was 21.9 (IQR, 19.7–23.6) kg/m^2^. Overall, 40 (43%) patients had GERD, 61 (65.6%) had hiatal hernia, and 64 (68.8%) had Barrett's esophagus. Further, 49 (52.7%) patients had a history of alcohol consumption, and 63 (67.7%) had a history of smoking. Endoscopic resection was performed for 47 lesions (50.5%), of which 15 (16.1%) subsequently required surgical resection. The remaining 46 lesions (49.5%) were treated using surgical resection. The median tumor size was 22 (IQR, 16–31) mm. In terms of location, tumors were almost evenly distributed between the esophageal and gastric sides (50.5% and 49.5%, respectively), and most lesions (73, 78.5%) were located on the right side. Tumor circumference was <1/2 in 87 (93.5%) lesions and ≥1/2 in six (6.5%) lesions. The main macroscopic type was “elevated” in 65 (69.9%) lesions, “flat‐depressed” in 28 (30.1%) lesions, and “complex” in 49 (52.7%) lesions. Histopathological evaluation revealed an undifferentiated component in 31 (33.3%) lesions, and 62 (66.7%) lesions were differentiated only. The depth of invasion was classified as M in 37 (39.8%) and as SM in 56 (60.2%) cases.

**TABLE 1:** Clinicopathological characteristics of patients and lesions.

Patient characteristics	
**Patient, No**.	93
**Age, years (median [IQR])**	67 (58–74)
**Sex, *n* (%)**	
Male	83 (89.2)
Female	10 (10.8)
**BMI, kg/m^2^ (median [IQR])**	21.9 (19.7–23.6)
**GERD, *n* (%)**	
+	40 (43)
−	53 (57)
**Hiatal hernia, *n* (%)**	
+	61 (65.6)
−	32 (34.4)
**Barrett's esophagus, *n* (%)**	
+	64 (68.8)
−	29 (31.2)
***Lesion characteristics***	
**Resection, *n* (%)**	
Endoscopic	32 (34.4)
Surgical after endoscopic	15 (16.1)
Surgical	46 (49.5)
**Tumor size, mm (median [IQR])**	22 (16–31)
**Tumor location, *n* (%)**	
Esophageal side ≥ Gastric side	47 (50.5)
Gastric side > Esophageal side	46 (49.5)
**Tumor location, *n* (%)**	
Right side	73 (78.5)
Left side	20 (21.5)
**Tumor circumference, *n* (%)**	
<1/2	87 (93.5)
≥1/2	6 (6.5)
**Main macroscopic type, *n* (%)**	
Elevated lesion	65 (69.9)
Flat‐depressed lesion	28 (30.1)
**Macroscopic type, *n* (%)**	
Simple	44 (47.3)
Complex	49 (52.7)
**Invasion depth, *n* (%)**	
M	37 (39.8)
SM	56 (60.2)
**Undifferentiated components, *n* (%)**	
+	31 (33.3)
−	62 (66.7)

Abbreviations: BMI, body mass index; GERD, gastroesophageal reflux disease; IQR, interquartile range; M, mucosal; SM, submucosal.

### 3.2 Diagnostic Performance

Table 2 presents the diagnostic performances of CE and CE + EUS in terms of depth assessment. CE + EUS showed a significantly lower specificity than did CE alone (62.2%; 95% confidence interval [CI], 44.8–77.5 [23/37] vs. 78.4%; 95% CI, 61.8–90.2 [29/37], respectively; *p* = 0.041). In addition, CE and CE + EUS showed a sensitivity of 73.2% (95% CI, 59.7–84.2 [41/56]) vs. 71.4% (95% CI, 57.8–82.7 [40/56]; *p* > 0.99), respectively, and an accuracy of 75.3% (95% CI, 65.2–83.6 [70/93]) vs. 67.7% (95% CI, 57.3–77.1 [63/93]; *p* = 0.121), respectively.

**TABLE 2:** Diagnostic performance of conventional endoscopy (CE) and CE + endoscopic ultrasonography (CE + EUS) for submucosal (SM) invasion.

	CE	CE + EUS	*p‐*Value
**Sensitivity No. (% [95% CI])**	41/56 (73.2 [59.7–84.2])	40/56 (71.4 [57.8–82.7])	>0.99
**Specificity No. (% [95% CI])**	29/37 (78.4 [61.8–90.2])	23/37 (62.2 [44.8–77.5])	0.041^a^
**Accuracy No. (% [95% CI])**	70/93 (75.3 [65.2–83.6])	63/93 (67.7 [57.3–77.1])	0.121

Abbreviations: CE, conventional endoscopy; CI, confidence interval; EUS, endoscopic ultrasonography; SM, submucosal.

*p*‐Values were calculated using McNemar's test.

^a^ *p* < 0.05 was considered statistically significant.

### 3.3 Misdiagnosed Patient Characteristics

Table 3 presents the clinicopathological characteristics of patients with invasion depth misdiagnosed using CE and CE + EUS. Although the difference in overall misdiagnosis rates was not statistically significant, the addition of EUS was associated with a 7.6% increase in misdiagnosis rate [24.7% (23/93) vs. 32.3% (30/93), *p* = 0.121]. The clinicopathological characteristics showed significant differences and increases in misdiagnoses with the addition of EUS in cases of lesions on the esophageal side [25.5% (12/47) vs. 42.6% (20/47), p = 0.027], elevated lesions [15.4% (10/65) vs. 29.2% (19/65), *p* = 0.008], complex lesions [16.3% (8/49) vs. 32.7% (16/49), *p* = 0.013], and lesions with hiatal hernia [19.7% (12/61) vs. 31.1% (19/61), *p* = 0.039]. Furthermore, to examine the clinical factors associated with overdiagnosis and underdiagnosis, we performed separate subgroup analyses for each group (Table 4). However, no clinicopathological factors were significantly associated with either overdiagnosis or underdiagnosis.

**TABLE 3:** Clinicopathological characteristics of misdiagnosed patients.

	Patients misdiagnosed, No./total No. (%)		
Variable	CE	CE plus EUS	*p‐*Value
**All cancers, *n* (%)**	23/93 (24.7)	30/93 (32.3)	0.121
**Tumor size, *n* (%)**			
≤2	10/45 (22.2)	15/45 (33.3)	0.074
>2	13/48 (27.1)	15/48 (31.3)	0.752
**Location of lesion, *n* (%)**			
Esophageal side ≥ Gastric side	12/47 (25.5)	20/47 (42.6)	0.027^a^
Gastric side > Esophageal side	11/46 (23.9)	10/46 (21.7)	>0.99
**Tumor location, *n* (%)**			
Right side	19/73 (26)	23/73 (31.5)	0.386
Left side	4/20 (20)	7/20 (35)	0.248
**Tumor circumference, *n* (%)**			
<1/2	21/87 (24.1)	27/87 (31)	0.181
≥1/2	2/6 (33.3)	3/6 (50)	>0.99
**Macroscopic type, *n* (%)**			
Elevated lesion	10/65 (15.4)	19/65 (29.2)	0.008^a^
Flat‐depressed lesion	13/28 (46.4)	11/28 (39.3)	0.683
**Macroscopic type, *n* (%)**			
Simple	15/44 (34.1)	14/44 (31.8)	>0.99
Complex	8/49 (16.3)	16/49 (32.7)	0.013^a^
**Undifferentiated components, *n* (%)**			
+	11/31 (35.5)	12/31 (38.7)	0.114
−	12/62 (19.4)	18/62 (29)	0.109
**GERD, *n* (%)**			
+	11/40 (27.5)	12/40 (30)	>0.99
−	12/53 (22.6)	18/53 (34)	0.114
**Hiatal hernia, *n* (%)**			
+	12/61 (19.7)	19/61 (31.1)	0.039^a^
−	11/32 (34.4)	11/32 (34.4)	>0.99

Abbreviations: CE, conventional endoscopy; EUS, endoscopic ultrasonography; GERD, gastroesophageal reflux disease.

*p*‐Values were calculated using McNemar's test.

^a^ *p* < 0.05 was considered statistically significant.

**TABLE 4:** Clinicopathological characteristics of overdiagnosed and underdiagnosed patients.

	Patients overdiagnosed, No./total No. (%)			Patients underdiagnosed, No./total No. (%)		
Variable	CE	CE plus EUS	*p‐*Value	CE	CE plus EUS	*p‐*Value
**All cancers, *n* (%)**	8/49 (16.3)	14/54 (25.4)	0.336	15/44 (34.1)	16/39 (41.0)	0.650
**Tumor size, *n* (%)**						
≤2	5/21 (23.8)	10/28 (35.7)	0.533	5/24 (20.8)	8/25 (32.0)	0.520
>2	3/28 (10.7)	4/26 (15.4)	0.699	10/20 (50.0)	8/14 (57.1)	0.738
**Location of lesion, *n* (%)**						
Esophageal side ≥ Gastric side	4/20 (20.0)	9/22 (40.9)	0.190	8/27 (29.6)	11/25 (44.0)	0.389
Gastric side > Esophageal side	4/29 (13.8)	5/32 (15.6)	>0.99	7/17 (41.2)	5/14 (35.7)	> 0.99
**Tumor location, *n* (%)**						
Right side	5/38 (13.2)	11/44 (25.0)	0.264	14/35 (40.0)	13/29 (44.8)	0.801
Left side	3/11 (27.3)	3/10 (30.0)	>0.99	1/9 (11.1)	3/10 (30.0)	0.582
**Tumor circumference, *n* (%)**						
<1/2	7/46 (15.2)	13/50 (26.0)	0.218	14/41 (34.1)	15/37 (40.5)	0.642
≥1/2	1/3 (33.3)	1/4 (25.0)	> 0.99	1/3 (33.3)	1/2 (50.0)	>0.99
**Macroscopic type, *n* (%)**						
Elevated lesion	6/32 (18.8)	9/35 (25.7)	0.566	3/23 (13.0)	5/20 (25.0)	0.440
Flat‐depressed lesion	2/17 (11.8)	5/19 (26.3)	0.408	12/21 (57.1)	11/19 (57.9)	>0.99
**Macroscopic type, *n* (%)**						
Simple	3/15 (20.0)	7/22 (31.8)	0.481	12/29 (41.4)	8/22 (36.4)	0.778
Complex	5/34 (14.7)	7/32 (21.9)	0.532	3/15 (20.0)	8/17 (47.1)	0.147
**Undifferentiated components, *n* (%)**						
+	2/18 (11.1)	3/18 (16.7)	0.669	9/14 (64.3)	10/15 (66.7)	>0.99
−	6/31 (19.4)	11/36 (30.6)	0.401	6/30 (20.0)	6/24 (25.0)	0.748
**GERD, *n* (%)**						
+	7/24 (29.2)	10/27 (37.0)	0.767	4/16 (25.0)	3/13 (23.1)	> 0.99
−	1/25 (4.0)	4/27 (14.8)	0.352	11/28 (39.3)	13/26 (50.0)	0.584
**Hiatal hernia, *n* (%)**						
+	3/31 (9.7)	7/32 (21.9)	0.302	9/30 (30.0)	12/29 (41.4)	0.422
−	5/18 (27.8)	7/22 (31.8)	>0.99	6/14 (42.9)	4/10 (40.0)	>0.99

Abbreviations: CE, conventional endoscopy; EUS, endoscopic ultrasonography; GERD, gastroesophageal reflux disease.

*p*‐Values were calculated using Fisher's exact test.

## 4 Discussion

Accurate evaluation of the depth of SM invasive cancer is crucial for appropriate treatment decisions for EGJA [19]. In the present study, we conducted a comparative analysis of the diagnostic performance of CE alone and CE + EUS for EGJA depth. However, our findings did not demonstrate any additional diagnostic value associated with the addition of EUS.

EUS is used as a diagnostic modality for tumor invasion depth in gastrointestinal cancers, and its accuracy in the upper gastrointestinal region has been previously reported [20, 21, 22, 23]. For the diagnosis of M or SM depth, meta‐analyses reported a sensitivity of 0.87 and a specificity of 0.94 for EUS in esophageal squamous cell carcinoma (SCC) and 0.87 and 0.75 for EUS in gastric cancer [20]. Meanwhile, the sensitivity and specificity of EUS in esophagogastric junction cancer were reported to be 0.59 and 0.69, respectively [21]. Furthermore, studies on Barrett's esophageal cancer have shown that EUS is less effective in diagnosing the depth of esophageal SCC and gastric cancers outside of the esophagogastric junction [22, 23]. Regarding the additive effect of EUS, a multi‐center study reported increased rates of overdiagnosis, with no improvement in diagnostic accuracy for esophageal SCC [24]. However, there are few studies on EGJA cases [12, 21].

In the present study, the addition of EUS to CE did not provide added value for depth assessment. Moreover, there was a significant increase in the number of cases with a misdiagnosed depth when CE + EUS was performed, CE for lesions located on the esophageal side, elevated lesions, complex lesions, and lesions associated with hiatal hernia. In addition, no significant clinicopathological factors were identified as risk factors for overdiagnosis or underdiagnosis, which may be partly attributable to the limited sample size.

The assessment of the layers at the esophagogastric junction poses challenges because of limitations such as inadequate extension provided by the lower esophageal sphincter and difficulties in achieving sufficient inflation, even with the use of de‐aerated water injection. In addition, the application of ultrasound in a vertical orientation becomes problematic when the esophageal wall is contracted. Furthermore, factors like the presence of endemic esophageal glands and vessels, mucosal fibrosis resulting from gastric acid reflux, and the sparse distribution of the lamina muscularis mucosa contribute to an increased likelihood of misdiagnosis when using EUS. In general, the submucosal layer of an elevated lesion may be difficult to delineate using a miniature probe owing to the thickness of the tumor [25]. Radial scanning endosonography can be employed for this purpose; however, due to the limited accumulation of water in the esophagogastric junction, utilizing the balloon method in conjunction with radial scanning endosonography may exert pressure on the layers, thereby elevating the risk of misdiagnosis, particularly in cases of overdiagnosis.

Additionally, achieving a comprehensive delineation of the entire lesion using EUS can be challenging in cases of macroscopic complex‐type lesions. When compared to macroscopic simple‐type lesions, complex‐type lesions exhibit a significantly higher likelihood of being SM invasive cancer and tend to pose relatively greater challenges in achieving accurate diagnosis [16]. In cases with a hiatal hernia, the hernia makes fluid retention difficult and the reflex stronger, thereby making probe positioning difficult; this may be a reason for the increased risk of misdiagnosis when using EUS. In previous studies, a tumor diameter of >5 cm was a significant factor for lower positive diagnosis rates [26]. In the present study, misdiagnosis tended to be more common with relatively large lesions ≥2 cm.

In recent years, certain findings have been presented regarding conventional and IEE for EGJA depth assessment. Oda et al. reported that cancer with SM invasion is characterized by a larger lesion size compared to M invasion, has more erythematous lesions, and is more likely to have macroscopically elevated (0‐I) and complex lesions than superficial ones [16]. Additionally, in recent years, an uneven surface and the presence of subsquamous extension have been reported as indicators of SM invasion or deeper invasion [17]. Yoshizawa et al. defined caliber variation (CV) in magnifying endoscopy as dilated irregular blood vessels with a diameter more than twice the diameter of capillaries in the superficial layer of the mucosa; in addition, the study reported that CV positivity was significantly higher in cancer with deep SM invasion [27]. With increasing diagnostic accuracy of CE, performing preoperative EUS, which does not have high diagnostic accuracy, may be less important owing to the associated increased rates of misdiagnosis. In contrast, the accuracy of EUS between cancers muscularis propria (MP) or deeper (T3/T4) and M‐SM (T1/T2) cancers is high in the esophagogastric junction [28]. Therefore, supplementary EUS may be useful for diagnosis when CE predicts it to be MP or deeper.

This study had several limitations. First, it was a retrospective, single‐center study, and not all EGJA cases underwent EUS, which may have introduced selection bias. To assess this potential effect, we retrospectively calculated the diagnostic performance of CE alone in 375 EGJA cases during the same period in which preoperative depth assessment was performed without EUS. The sensitivity, specificity, and accuracy were 70.3%, 84.4%, and 79.2%, respectively, with no statistically significant differences observed when compared to CE performance in the present study cohort (Table S1). Second, the diagnostic performance of endoscopy was based on endoscopic reports at the time of examination and was not centrally reviewed. Third, differences in histopathological assessment between surgically and endoscopically resected specimens may have affected the accuracy of the reference standard, as surgical specimens are typically sectioned at 5‐mm intervals, potentially leading to underestimation of submucosal invasion compared with 2‐mm slices in endoscopic specimens. Finally, the sample size of this study was limited, which may have affected the ability to detect significant risk factors for overdiagnosis and underdiagnosis.

In conclusion, the addition of EUS to CE reduced the ability to correctly identify tumors confined to the mucosa in early‐stage EGJA. These findings suggest that the clinical utility of EUS in this setting is limited, as it may increase the risk of overdiagnosis and lead to unnecessary overtreatment.

## Author Contributions

**Keita Suzuki** contributed to the conceptualization of the study and the drafting of the manuscript. **Yohei Ikenoyama** contributed to the conceptualization of the study, drafting of the manuscript, and critical revision of the manuscript for important intellectual content. **Ken Namikawa**, **Yoshitaka Tokai**, **Shoichi Yoshimizu**, **Yusuke Horiuchi**, **Akiyoshi Ishiyama**, **Toshiyuki Yoshio**, **Toshiaki Hirasawa**, **Manabu Takamatsu**, **Takahisa Matsuda**, and **Junko Fujisaki** contributed to the critical revision of the manuscript for important intellectual content. All authors approved the final manuscript.

## Ethics Statement

The study was conducted in accordance with the principles of the Declaration of Helsinki, and the study protocol was approved by the Ethics Committee of the Cancer Institute Hospital, Japanese Foundation for Cancer Research (no. 2020‐1324).

## Consent

Informed consent was obtained from all patients in the study using the opt‐out method.

## Conflicts of Interest

The authors declare no conflicts of interest.

## Clinical Trial Registration

N/A

## Supporting information




**TABLE S1** Diagnostic performance of CE (study cohort) and CE only (no EUS, contemporaneous cohort) for SM invasion.

## Data Availability

The data are not available for public access because of patient privacy concerns, but are available from the corresponding author on reasonable request.

## References

[1] K. Matsuno , R. Ishihara , M. Ohmori , et al., “Time Trends in the Incidence of Esophageal Adenocarcinoma, Gastric Adenocarcinoma, and Superficial Esophagogastric Junction Adenocarcinoma,” Journal of Gastroenterology 54 (2019): 784–791. 30927083 10.1007/s00535-019-01577-7

[2] C. Kusano , T. Gotoda , C. J. Khor , et al., “Changing Trends in the Proportion of Adenocarcinoma of the Esophagogastric Junction in a Large Tertiary Referral Center in Japan,” Journal of Gastroenterology and Hepatology 23 (2008): 1662–1665. 19120859 10.1111/j.1440-1746.2008.05572.x

[3] T. Nishi , H. Makuuchi , S. Ozawa , H. Shimada , and O. Chino , “The Present Status and Future of Barrett's Esophageal Adenocarcinoma in Japan,” Digestion 99 (2019): 185–190. 30481763 10.1159/000490508

[4] T. Nishi , H. Makuuchi , H. Shimada , et al., “Prognosis of Barrett's Cancer,” Clinical Gastroenterol 22 (2007): 111–205.

[5] J. Aida , T. Ishizaki , T. Arai , and K. Takubo , “members of the Japan Research Society for Early Esophageal Cancer and Chromoendoscopy. Prognostication of Superficial Barrett's Carcinoma: A Japanese Multicenter Study,” Human Pathology 76 (2018): 156–166. 29534888 10.1016/j.humpath.2018.03.001

[6] S. Yoshinaga , T. Gotoda , C. Kusano , I. Oda , K. Nakamura , and R. Takayanagi , “Clinical Impact of Endoscopic Submucosal Dissection (ESD) for Superficial Adenocarcinoma Located at the Esophagogastric Junction (EGJ),” Gastrointestinal Endoscopy 67 (2008): 202–209. 18226681 10.1016/j.gie.2007.09.054

[7] I. Hoshino , H. Gunji , F. Ishige , et al., “Surgical Treatment Strategy for Esophagogastric Junction Cancers Based on the Tumor Diameter,” BMC Surgery [Electronic Resource] 19 (2019): 152. 31651313 10.1186/s12893-019-0614-5 PMC6814119

[8] T. Rösch , R. Lorenz , K. Zenker , et al., “Local Staging and Assessment of Resectability in Carcinoma of the Esophagus, Stomach, and Duodenum by Endoscopic Ultrasonography,” Gastrointestinal Endoscopy 38 (1992): 460–467. 1511822 10.1016/s0016-5107(92)70477-5

[9] Y. Murata , S. Suzuki , M. Ohta , et al., “Small Ultrasonic Probes for Determination of the Depth of Superficial Esophageal Cancer,” Gastrointestinal Endoscopy 44 (1996): 23–28. 8836712 10.1016/s0016-5107(96)70224-9

[10] Y. Tsujii , M. Kato , T. Inoue , et al., “Integrated Diagnostic Strategy for the Invasion Depth of Early Gastric Cancer by Conventional Endoscopy and EUS,” Gastrointestinal Endoscopy 82 (2015): 452–459. 25841580 10.1016/j.gie.2015.01.022

[11] T. Nagahama , K. Yao , K. Imamura , et al., “Diagnostic Performance of Conventional Endoscopy in the Identification of Submucosal Invasion by Early Gastric Cancer: The “Non‐extension Sign” as a Simple Diagnostic Marker,” Gastric Cancer 20 (2017): 304–313. 27165641 10.1007/s10120-016-0612-6

[12] A. May , E. Günter , F. Roth , et al., “Accuracy of Staging in Early Oesophageal Cancer Using High Resolution Endoscopy and High Resolution Endosonography: A Comparative, Prospective, and Blinded Trial,” Gut 53 (2004): 634–640. 15082579 10.1136/gut.2003.029421 PMC1774048

[13] Japanese Gastric Cancer Association , “Japanese Classification of Gastric Carcinoma: 3^rd^ English Edition,” Gastric Cancer 14 (2011): 101–112. 21573743 10.1007/s10120-011-0041-5

[14] J. R. Siewert and H. J. Stein , “Classification of Adenocarcinoma of the Oesophagogastric Junction,” British Journal of Surgery 85 (1998): 1457–1459. 9823902 10.1046/j.1365-2168.1998.00940.x

[15] Japan Esophageal Society , “Japanese Classification of Esophageal Cancer, 11th edition: Part I,” Esophagus 14 (2017): 1–36. 28111535 10.1007/s10388-016-0551-7 PMC5222932

[16] I. Oda , S. Abe , C. Kusano , et al., “Correlation Between Endoscopic Macroscopic Type and Invasion Depth for Early Esophagogastric Junction Adenocarcinomas,” Gastric Cancer 14 (2011): 22–27. 21274736 10.1007/s10120-011-0001-0

[17] K. Takada , Y. Yabuuchi , Y. Yamamoto , et al., “Predicting the Depth of Superficial Adenocarcinoma of the Esophagogastric Junction,” Journal of Gastroenterology and Hepatology 37 (2022): 363–370. 34820917 10.1111/jgh.15741

[18] “The Paris Endoscopic Classification of Superficial Neoplastic Lesions: Esophagus, Stomach, and Colon: November 30 to December 1, 2002,”. Gastrointestinal Endoscopy 58 (2003): S3–43. 14652541 10.1016/s0016-5107(03)02159-x

[19] Y. Shoji , K. Koyanagi , K. Kanamori , et al., “Current Status and Future Perspectives for the Treatment of Resectable Locally Advanced Esophagogastric Junction Cancer: A Narrative Review,” World Journal of Gastroenterology 29 (2023): 3758–3769. 37426325 10.3748/wjg.v29.i24.3758 PMC10324534

[20] R. Ishihara , K. Goda , and T. Oyama , “Endoscopic Diagnosis and Treatment of Esophageal Adenocarcinoma: Introduction of Japan Esophageal Society Classification of Barrett's Esophagus,” Journal of Gastroenterology 54 (2019): 1–9. 29961130 10.1007/s00535-018-1491-x PMC6314977

[21] R. Dhupar , R. D. Rice , A. M. Correa , et al., “Endoscopic Ultrasound Estimates for Tumor Depth at the Gastroesophageal Junction Are Inaccurate: Implications for the Liberal Use of Endoscopic Resection,” Annals of Thoracic Surgery 100 (2015): 1812–1816. 26233274 10.1016/j.athoracsur.2015.05.038

[22] T. Thomas , D. Gilbert , P. V. Kaye , I. Penman , G. P. Aithal , and K. Ragunath , “High‐resolution Endoscopy and Endoscopic Ultrasound for Evaluation of Early Neoplasia in Barrett's Esophagus,” Surgical Endoscopy 24 (2010): 1110–1116. 19915911 10.1007/s00464-009-0737-3

[23] J. O. Fernández‐Sordo , V. J. Konda , J. Chennat , et al., “Is Endoscopic Ultrasound (EUS) Necessary in the Pre‐therapeutic Assessment of Barrett's Esophagus With Early Neoplasia?” Journal of Gastrointestinal Oncology 3 (2012): 314–321. 23205307 10.3978/j.issn.2078-6891.2012.038 PMC3492480

[24] R. Ishihara , J. Mizusawa , R. Kushima , et al., “Assessment of the Diagnostic Performance of Endoscopic Ultrasonography After Conventional Endoscopy for the Evaluation of Esophageal Squamous Cell Carcinoma Invasion Depth,” JAMA Network Open 4 (2021): e2125317. 34524432 10.1001/jamanetworkopen.2021.25317 PMC8444025

[25] K. Akashi , H. Yanai , J. Nishikawa , et al., “Ulcerous Change Decreases the Accuracy of Endoscopic Ultrasonography Diagnosis for the Invasive Depth of Early Gastric Cancer,” International Journal of Gastrointestinal Cancer 37 (2006): 133–138. 18080789 10.1007/s12029-007-9004-9

[26] P. A. Heeren , H. L. van Westreenen , G. J. Geersing , H. M. van Dullemen , and J. T. Plukker , “Influence of Tumor Characteristics on the Accuracy of Endoscopic Ultrasonography in Staging Cancer of the Esophagus and Esophagogastric Junction,” Endoscopy 36 (2004): 966–971. 15520913 10.1055/s-2004-825956

[27] N. Yoshizawa , J. Fujisaki , M. Omae , M. Igarashi , S. Tanabe , and W. Koizumi , “Study of the Invasion Depth Diagnosis of superficial Barrett's Esophageal Adenocarcinoma‐identification of Large Vessels by Magnifying Endoscopy Is Useful for the Diagnosis of Sub Mucosal Cancer,” Advanced Research in Gastroenterology & Hepatology 2 (2016): 555576.

[28] S. Kelly , K. M. Harris , E. Berry , et al., “A Systematic Review of the Staging Performance of Endoscopic Ultrasound in Gastro‐oesophageal Carcinoma,” Gut 49 (2001): 534–539. 11559651 10.1136/gut.49.4.534 PMC1728456

