# Supplementary material for: Evaluating the Additional Value of Endoscopic Ultrasonography for Depth Assessment of Esophagogastric Junction Adenocarcinoma
Source: DEN Open. 2025 Sep 30;6(1):e70215. doi: 10.1002/deo2.70215 (PMC12481211; doi:10.1002/deo2.70215)
Supplement: Supplementary file 1 — TABLE S1 Diagnostic performance of CE (study cohort) and CE only (no EUS, contemporaneous cohort) for SM invasion. [file DEO2-6-e70215-s001.docx]

**Supplementary Table 1.** Diagnostic performance of CE (study cohort) and CE only (no EUS, contemporaneous cohort) for SM invasion

|  | **CE (study cohort)** | **CE only (no EUS)** | ***P-value*** |
| --- | --- | --- | --- |
| **Sensitivity No. (% [95% CI])** | 41/56  (73.2 [59.7–84.2]) | 97/138  (70.3 [62.2–77.3]) | 0.730 |
| **Specificity No. (% [95% CI])** | 29/37  (78.4 [61.8–90.2]) | 200/237  (84.4 [79.2–88.5]) | 0.346 |
| **Accuracy No. (% [95% CI])** | 70/93  (75.3 [65.2–83.6]) | 297/375  (79.2 [74.8–83.0]) | 0.402 |

CE, conventional endoscopy; CI, confidence interval; EUS, endoscopic ultrasonography; SM, submucosal

P-values were calculated using Fisher’s exact test. P < 0.05 was considered statistically significant.
